# Pericardial Calcification Following Acute Myopericarditis After Initial CHOP Treatment for Mantle Cell Lymphoma: A Case Report

**DOI:** 10.7759/cureus.88634

**Published:** 2025-07-23

**Authors:** Keishi Miyazawa, Yukari Shirasugi, Eriya Imai, Jun Tanaka, Joe Toda, Kensuke Narukawa, Yurika Mitsui, Tsuyoshi Takahashi

**Affiliations:** 1 Department of Hematology, Mitsui Memorial Hospital, Tokyo, JPN; 2 Department of Anesthesiology, Saiseikai Yokohamashi Tobu Hospital, Yokohama, JPN; 3 Division of Anesthesia, Mitsui Memorial Hospital, Tokyo, JPN; 4 Department of Social Medical Sciences, Graduate School of Medicine, International University of Health and Welfare, Tokyo, JPN; 5 Department of Cardiology, Mitsui Memorial Hospital, Tokyo, JPN; 6 Department of Diagnostic Radiology, Mitsui Memorial Hospital, Tokyo, JPN

**Keywords:** acute myopericarditis, b-cell lymphoma, calcification of the pericardium, cardiooncology, chemotherapy associated cardiotoxicity, chop chemotherapy, mantle cell lymphoma (mcl), tumor-lysis syndrome

## Abstract

A 56-year-old woman with B-cell lymphoma underwent chemotherapy with cyclophosphamide, doxorubicin, vincristine, and prednisolone (CHOP). Within 30 hours, the patient developed acute cardiotoxicity, characterized by elevated cardiac enzymes and ST-segment and T-wave (ST-T) changes on electrocardiogram (ECG), despite normal echocardiographic findings. Both ST elevations and enzyme levels spontaneously improved the following day. One week later, computed tomography (CT) revealed a newly developed high-density area on the pericardium, suggestive of early calcific deposition, which was confirmed to have progressed on follow-up imaging. While anthracycline-related cardiovascular toxicities are well documented, this case demonstrates a rare and rapid evolution from acute myopericarditis to early pericardial calcification following initial CHOP therapy. This rare presentation of early-onset cardiotoxicity in a patient with B-cell lymphoma is discussed with reference to the existing literature.

## Introduction

Chemotherapy regimens such as CHOP (cyclophosphamide, doxorubicin, vincristine, prednisolone) or R-CHOP (CHOP plus rituximab) remain the standard first-line treatments for non-Hodgkin lymphoma (NHL) [1,2]. While effective, these regimens are associated with a broad spectrum of cardiovascular toxicities. In particular, the alkylating agent cyclophosphamide and the anthracycline doxorubicin are well known to cause both acute and chronic cardiotoxic effects. However, the incidence, timing, and clinical characteristics of chemotherapy-related cardiovascular complications remain incompletely understood [2]. Given that CHOP is expected to remain a cornerstone of frontline treatment for NHL [1,2], case reports documenting cardiovascular complications are valuable for enhancing clinical awareness and guiding cardiotoxicity monitoring and management strategies in this population.

Here, we report a patient who developed acute electrocardiographic abnormalities and elevated cardiac biomarkers just 30 hours after the first administration of CHOP, findings highly suggestive of an acute cardiac event. Remarkably, this was followed by the rapid onset of pericardial calcification within one week, a rare and atypical manifestation of chemotherapy-induced cardiotoxicity.

This case was previously presented as a meeting abstract at the 19th Kanto Branch Meeting of the Japanese Society of Hematology on July 15, 2023.

## Case presentation

**Figure 1:**
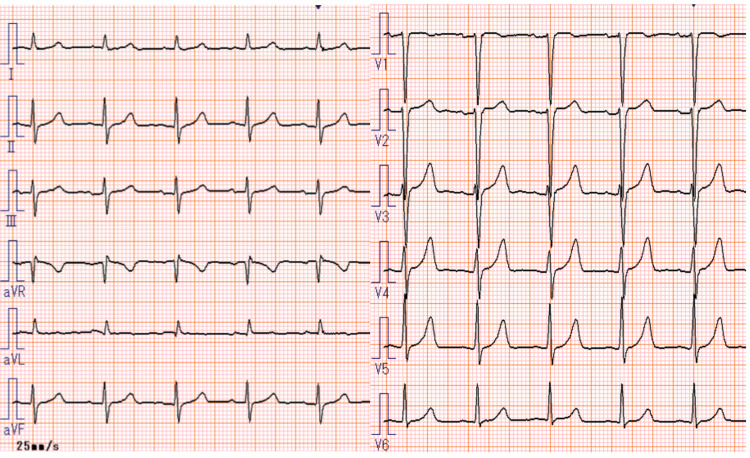
ECG on the day of admission. ECG: electrocardiogram. Normal sinus rhythm with no ST-T changes observed.

A 56-year-old Japanese woman presented with lower right abdominal and back pain and was referred to our hospital. The patient had no prior history of cardiovascular disease, and the electrocardiogram (ECG) obtained on the day of admission showed no ST-segment and T-wave (ST-T) changes (Figure 1). A computed tomography (CT) scan revealed multiple enlarged lymph nodes in the para-aortic region extending to the pelvic cavity and bilateral inguinal areas, raising suspicion of malignant lymphoma. Biopsies of both inguinal lymph nodes were performed but yielded inconclusive immunohistological findings. To obtain a definitive pathological diagnosis, a laparoscopic biopsy of the para-aortic lymph nodes was conducted. However, on the same day, the patient developed tumor lysis syndrome (TLS), resulting in renal dysfunction and pulmonary congestion, and was admitted to the intensive care unit.

Although a definitive diagnosis had not yet been established, the patient’s deteriorating condition prompted the initiation of prednisolone therapy (50 mg/day; 1 mg/kg/day) as empiric treatment for suspected lymphoma. Due to TLS-associated pulmonary edema and renal dysfunction, the patient required orotracheal intubation with intermittent positive-pressure ventilation and continuous hemodiafiltration. An interim pathological report from the para-aortic lymph nodes revealed a proliferation of medium- to large-sized atypical lymphoid cells that were CD20 and bcl-2 positive, but both CD5 and CD10 were negative by immunohistochemistry. Flow cytometry demonstrated the presence of CD19+/CD20+ cells with kappa light chain restriction, strongly suggestive of diffuse large B-cell lymphoma. Consequently, full-dose CHOP chemotherapy (cyclophosphamide 750 mg/m^2^, doxorubicin 50 mg/m^2^, vincristine 1.4 mg/m^2^, and prednisolone 100 mg/body) was initiated.

**Figure 2:**
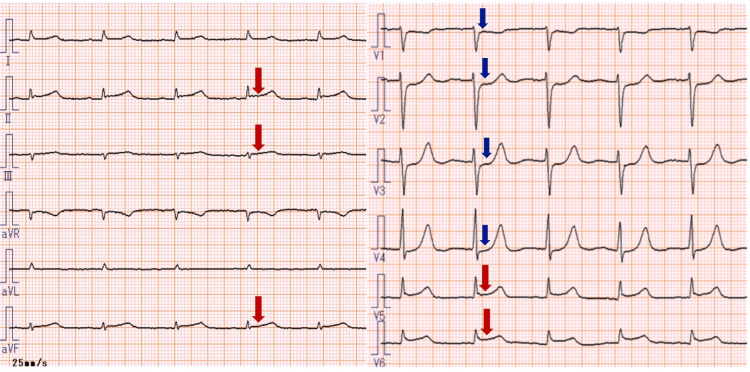
Electrocardiographic changes 30 hours after initiation of CHOP therapy. ST-segment elevations are seen in leads II, III, aVF, V5, and V6 (red arrows), accompanied by reciprocal ST-segment depressions in precordial leads V1 to V4 (blue arrows). CHOP therapy: Chemotherapy regimen consisting of cyclophosphamide, doxorubicin, vincristine, and prednisolone.

**Figure 3:**
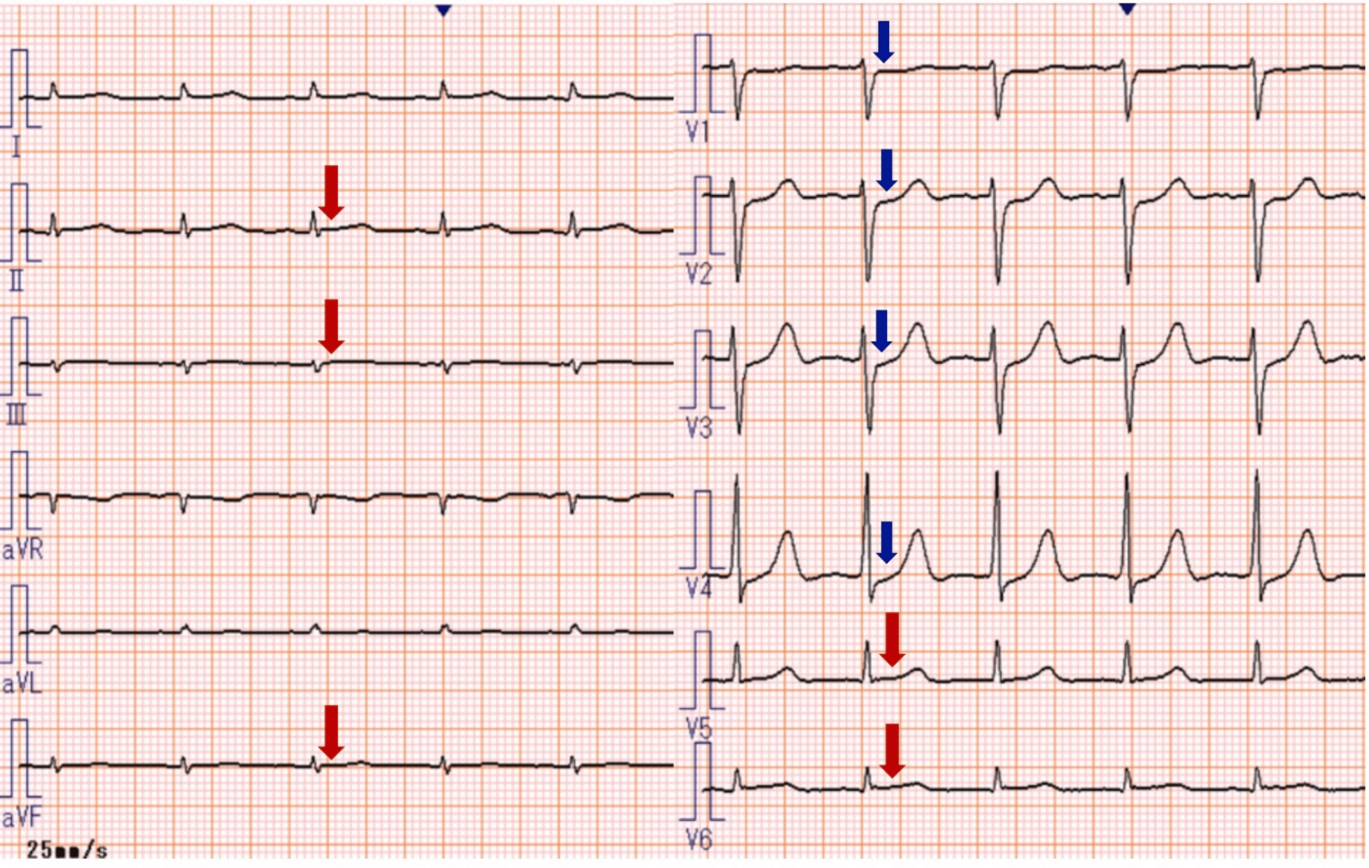
Electrocardiographic changes three days after initiation of CHOP therapy. ST-segment elevations in leads II, III, aVF, V5, and V6 (red arrows) have resolved, and reciprocal ST-segment depressions in precordial leads V1 to V4 (blue arrows) have improved. No abnormal Q waves were observed. CHOP therapy: Chemotherapy regimen consisting of cyclophosphamide, doxorubicin, vincristine, and prednisolone.

Approximately 30 hours after CHOP initiation, the patient exhibited sudden ECG changes characterized by ST-segment elevation in leads II, III, aVF, V5, and V6, along with reciprocal ST-segment depressions in precordial leads V1 to V4 (Figure 2). These findings were classified as Grade 2 according to the Common Terminology Criteria for Adverse Events (CTCAE) version 5.0. (National Cancer Institute). Bedside transthoracic echocardiography (TTE) showed no regional wall motion abnormalities. However, laboratory testing revealed significant elevations in myocardial biomarkers. The serial changes in myocardial biomarkers and electrolyte levels are summarized in Table 1. During the cardiac event, the patient was intubated and sedated, making it impossible to assess the presence or absence of chest pain. All myocardial biomarkers peaked approximately 38 hours after CHOP initiation and gradually declined thereafter. Two days after the cardiac event (i.e., three days after CHOP initiation), ST-segment elevations in leads II, III, aVF, V5, and V6 had resolved, and reciprocal ST-segment depressions in leads V1 to V4 had improved (Figure 3).

**Table 1:** Serial changes in myocardial biomarkers and electrolyte levels after initiation of CHOP therapy. CK: creatinine kinase; CHOP therapy: chemotherapy regimen consisting of cyclophosphamide, doxorubicin, vincristine, and prednisolone; CTCAE: Common Terminology Criteria for Adverse Events (National Cancer Institute); TnT: troponin T; NT-proBNP: N-terminal pro–B-type natriuretic peptide; IP: inorganic phosphorus.

Laboratory test	Reference range	Unit	Two hours after CHOP initiation/CTCAE v5.0 Grade	30 Hours after CHOP initiation/CTCAE v5.0 Grade	38 Hours after CHOP initiation (peak)/CTCAE v5.0 Grade	Three days after CHOP initiation
							CK	41-153	U/L	35	646/Grade 2	1021/Grade 3	283
							CK-MB	0-12	U/L	-	194/ NA	256/NA	-
Cardiac TnT	0-14	ng/L	-	1,340/Grade 3	2710/Grade 3	-
NT-proBNP	0-55	pg/mL	-	-	-	12,305
Potassium (K)	3.6-4.8	mmol/L	5.7/Grade 2	4.1	4.1	4.2
IP	2.7-4.6	mg/dL	6.2	7.6	7.5	6.7
Calcium (Ca)	8.8-10.1	mg/dL	10.2/Grade 1	9.1	8.8	8.7

**Figure 4:**
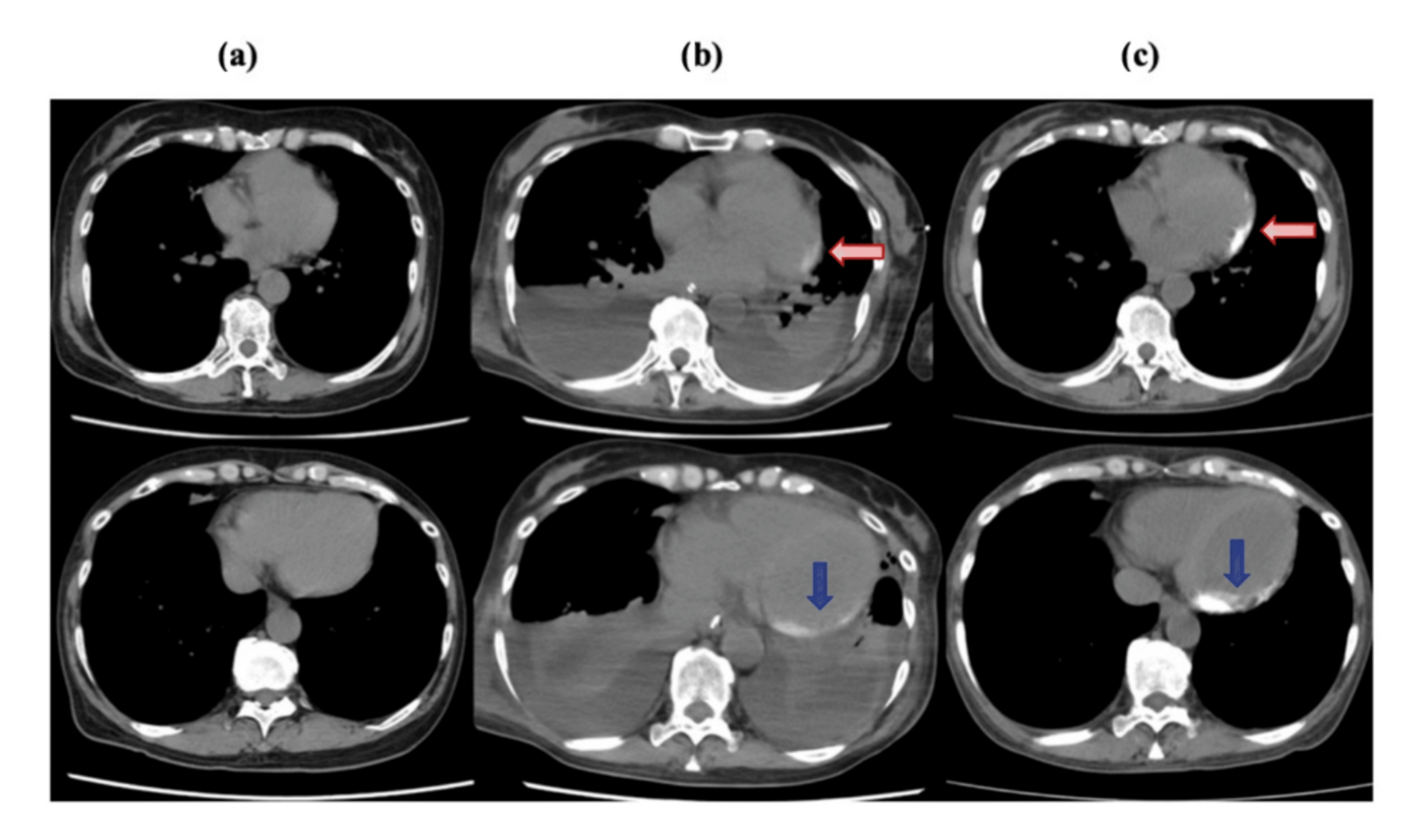
Newly developed high-density area on the pericardium observed on CT. (a) Thirty-five days prior to CHOP therapy: No abnormal high-density area observed. (b) One week after CHOP therapy: A newly developed high-density area appeared on the pericardium along the lateral (red arrow) and inferior walls (blue arrow) of the heart. (c) Two months after CHOP therapy: The previously noted high-density areas on the lateral (red arrow) and inferior (blue arrow) pericardium became more prominent, raising suspicion of pericardial calcification. CT: computed tomography.

A non-contrast CT scan performed one week later revealed a new high-density area on the pericardium overlying the lateral and inferior walls of the heart (Figure 4b). In the absence of prior cardiac disease or alternative causes, these findings were considered most consistent with chemotherapy-induced acute myopericarditis following initial CHOP treatment. The patient's condition gradually stabilized, and she eventually recovered from intensive care. 

The final pathological report of the para-aortic lymph nodes revealed bcl-6 negativity and cyclin-D1 positivity, with chromosomal analysis confirming t(11;14)(q13;q32), establishing a diagnosis of CD5-negative mantle cell lymphoma. As a result, the treatment regimen was changed to a BR regimen (bendamustine 90 mg/m^2^, rituximab 375 mg/m^2^) for subsequent chemotherapy cycles.

A follow-up non-contrast CT scan conducted two months after the cardiac event revealed further progression of the pericardial high-density area, with a CT value of 250 Hounsfield units, confirming pericardial calcification (Figure 4c). Coronary CT angiography ruled out coronary artery stenosis, while TTE demonstrated preserved left ventricular ejection fraction (LVEF; 58%) and no regional wall motion abnormalities. A comparison with echocardiography performed at the time of the ST-T changes revealed no significant alteration in LVEF and global longitudinal strain (GLS). 

## Discussion

Patients receiving CHOP chemotherapy for NHL are at risk for a spectrum of therapy-related cardiotoxic effects [2]. While reductions in LVEF and the development of heart failure are well-recognized manifestations of chemotherapy-induced cardiotoxicity, cancer therapies can affect the entire cardiovascular system [3,4]. The International Cardio-Oncology Society classifies chemotherapy-related cardiovascular toxicities into five major categories: (1) cardiac dysfunction, including cardiomyopathy and heart failure; (2) myocarditis; (3) vascular toxicity; (4) hypertension; and (5) arrhythmias, including QTc prolongation [5]. 

In the present case, sudden ECG changes were observed the day after the first administration of CHOP chemotherapy, characterized by ST-segment elevation in leads II, III, aVF, V5, and V6, with reciprocal ST-segment depressions in leads V1 to V4 (Figure 2). Concurrently, serum levels of myocardial biomarkers, including creatinine kinase (CK), CK-MB, and cardiac troponin T (TnT), were markedly elevated (Table 1), suggestive of acute myocardial injury, and initially raising concern for acute myocardial infarction (AMI). However, AMI was ruled out based on two key findings: TTE revealed no regional wall motion abnormalities, and coronary CT angiography showed no evidence of coronary artery stenosis.

Acute pericarditis often coexists with some degree of myocarditis [6], and cases with predominant pericardial involvement alongside myocardial injury are classified as myopericarditis, as defined by the European Society of Cardiology (ESC) [7]. The diagnosis of myopericarditis is based on fulfilling criteria for acute pericarditis, including chest pain, pericardial rubs, ST-segment elevation, and pericardial effusion, along with elevated cardiac biomarkers (troponin I/T or CK-MB), in the absence of new regional or global left ventricular dysfunction on echocardiography [7]. In our patient, intubation and sedation during the cardiac event precluded the assessment of chest pain. However, other diagnostic criteria were fulfilled, supporting a diagnosis of acute myopericarditis. Although endomyocardial biopsy remains the gold standard, it was not feasible due to the patient’s unstable condition.

The etiology of myopericarditis was most likely related to CHOP chemotherapy, particularly doxorubicin and cyclophosphamide [2,5,7,8], though other potential contributing factors cannot be entirely excluded. Acute myopericarditis may develop shortly after anthracycline administration [8], with known risk factors including pre-existing cardiac dysfunction, older age, concomitant chemotherapeutic agents, and hematologic malignancies, such as lymphoma [8]. Although evidence is limited, previous case reports suggest that renal impairment may exacerbate cyclophosphamide-associated cardiotoxicity, including myopericarditis [9]. Our patient, who required continuous hemodiafiltration due to TLS-associated acute kidney injury, may have been similarly predisposed.

A systematic review and meta-analysis of cardiovascular adverse events (CVAEs) in patients with NHL receiving first-line CHOP or R-CHOP therapy (77 studies; n=14,351) reported a pooled incidence of grade 3-4 CVAEs of 2.35% (95%CI: 1.81-2.93%), with a range from 0.0% to 15.1% [2]. Female sex and age over 65 were identified as independent risk factors [2]. Our patient, a woman under 65 with no prior cardiovascular history, developed grade 3 elevations in CK and TnT and was diagnosed with myopericarditis within 30 hours of CHOP initiation, an exceptionally rare and unpredictable adverse event.

An alternative explanation could involve subclinical cardiac infiltration by lymphoma prior to chemotherapy. Although initial echocardiography and CT scans did not show overt cardiac involvement, minor infiltration of the lateral and inferior myocardium or pericardium cannot be completely excluded. In such a scenario, CHOP chemotherapy, especially doxorubicin and cyclophosphamide, may have triggered localized tumor lysis, resulting in focal myocardial inflammation and subsequent pericardial calcification.

A non-contrast CT scan performed one week after the cardiac event revealed a new high-density area on the pericardium overlying the lateral and inferior walls of the heart. Follow-up imaging two months later showed increased density and extent, consistent with evolving pericardial calcification (Figure 4). The normal pericardium lacks calcium, and calcification typically arises secondary to prior inflammation, fibrosis, and necrosis [10]. Known etiologies of pericardial calcification include prior infections, malignancy, and rheumatologic or connective tissue disorders [10,11]. A systematic review of myocardial calcification also implicated sepsis, chronic kidney disease, and acute systemic inflammatory states such as TLS [12]. Pericardial calcification is usually detected incidentally on CT and is often asymptomatic [13]. The 2015 ESC guidelines recommended CT and/or cardiac magnetic resonance imaging as second-line imaging modalities in the diagnosis of pericarditis (Class I, Level of Evidence C) [7,11].

Remarkably, in our case, pericardial calcification developed within just one week, an unusually short interval. A similar case of rapidly progressive myocardial calcification associated with TLS in a patient with B-cell acute lymphoblastic leukemia (B-ALL) has been reported [14]. In that case, electrocardiographic ST elevations and biomarker elevation appeared within two to three hours after initiating prednisolone therapy, and the patient unfortunately died three days later. Autopsy revealed calcium deposits within myocardial fibers. It has been reported that calcium deposition in damaged cardiomyocytes can begin within days and progress over weeks to months [15]. In the B-ALL case, the authors hypothesized that hyperphosphatemia played a central role, with calcium-phosphate imbalance promoting metastatic calcification in necrotic cardiomyocytes [14]. These findings underscore the importance of considering metastatic calcification in the differential diagnosis of acute cardiac events, particularly in the context of TLS or other metabolic disturbances. In our case, although the patient underwent continuous hemodiafiltration, both serum inorganic phosphate and calcium levels remained persistently elevated following CHOP initiation, and no abrupt rise in serum phosphorus typical of TLS was observed (Table 1). However, it is conceivable that CHOP-induced myopericarditis created a localized inflammatory milieu conducive to calcium-phosphate precipitation, contributing to the unusually rapid development of pericardial calcification.

## Conclusions

This case highlights a rare occurrence of chemotherapy-induced myopericarditis with early-onset pericardial calcification following the initial administration of CHOP therapy. The unusually rapid development of calcification, within just one week, raises the possibility of an additional contribution from TLS. Although CHOP remains a widely used and generally well-tolerated regimen for NHL, this case underscores the importance of vigilant cardiac monitoring, even in patients without prior cardiovascular disease. Furthermore, the emergence of pericardial calcification raises concerns about potential progression to constrictive pericarditis, emphasizing the need for careful long-term cardiac surveillance in similar cases.
